# Serum Dickkopf-1 Level in Postmenopausal Females: Correlation with Bone Mineral Density and Serum Biochemical Markers

**DOI:** 10.1155/2013/460210

**Published:** 2013-06-27

**Authors:** Sahar Fathi Ahmed, Neveen Fouda, Amal Ahmed Abbas

**Affiliations:** ^1^Rheumatology and Rehabilitation Department, Faculty of Medicine, Ain Shams University, P.O. Box 11566, Abbassia Square, Cairo, Egypt; ^2^Clinical Pathology Department, Faculty of Medicine, Ain Shams University, P.O. Box 11566, Abbassia Square, Cairo, Egypt

## Abstract

*Objective*. To assess serum level of Dickkopf-1 in postmenopausal females and its correlation with bone mineral density and serum biochemical markers. *Methods*. Bone densitometry, serum Dickkopf-1, calcium, phosphorus, and alkaline phosphatase were done in sixty postmenopausal females. Patients were divided according to *T* score into osteoporosis (group I), osteopenia (group II), and normal bone mineral density that served as controls. *Results*. There was highly significant increase in serum Dickkopf-1 levels in postmenopausal females with abnormal *T* score versus controls (*P* < 0.001). Serum DKK-1 levels correlated negatively with both lumbar *T* score (*r* = −0.69, *P* < 0.001) and femur *T* score (*r* = −0.64, *P* < 0.001) and correlated positively with duration of menopause (*r* = 0.61, *P* < 0.001), while there was no significant correlation between serum levels of either calcium, phosphorus or alkaline phosphatase, and both serum Dickkopf-1 levels and the level of bone mineral density (*P* > 0.05). *Conclusion*. Postmenopausal females may suffer from osteoporosis as evidenced by bone densitometry. Postmenopausal women with significantly increased serum Dickkopf-1 had more significant osteoporosis. Prolonged duration of menopause and increased serum Dickkopf-1 are important risk factors for the development and severity of osteoporosis.

## 1. Introduction

Osteoporosis is a systemic skeletal disease characterized by low bone mass and microarchitectural deterioration of bone tissue, with a consequent increase in bone fragility [1]. It is a chronic progressive disease of multifactorial etiology and one of the most common metabolic bone diseases frequently recognized in postmenopausal women [2]. Osteoporosis is a preventable disease that can result in devastating physical, psychosocial, and economic consequences. Prevention and recognition of osteoporosis are first-line measures to lessen the impact of this condition [3].

 Recent evidence suggests that Dickkopf-1 (DKK-1), a soluble inhibitor of the Wnt pathway, may be an active player in several critical aspects of bone biology [4, 5]. The Wnt pathway was first described more than 20 years ago but its role in bone biology was only recently unraveled [6, 7]. The Wnt/*β*-catenin, also called “canonical” Wnt pathway (1 of the 4 Wnt signaling pathways), is a critical regulator of many aspects of bone and joint physiology [8].

 The Wnt/*β*-catenin pathway stimulates bone formation in several ways: (1) by stimulating osteoblast differentiation and activity and blocking the differentiation of mesenchymal cells toward chondrocytes or adipocytes; (2) by increasing the growth rate of osteoblasts and inhibiting their apoptosis; and, finally, (3) by inhibiting osteoclastogenesis [9]. So, Wnt pathway activation enhances bone mass not only by stimulating osteoblastogenesis but, at least to some extent, also by inhibiting osteoclastogenesis as well [10]. 

 Dickkopfs (DKKs) are secreted developmental regulators composed of two cysteine-rich domains. The amino terminal domain of DKK1 plays an inhibitory role on Wnt signalling by binding to the low-density lipoprotein receptor-related protein (LRP5/LRP6) component of the Wnt receptor complex [4]. So, enhanced Wnt signaling results in increased bone formation, whereas decreased signaling by DKK1 has the opposite effect [11].

 Therefore, we aimed to assess the serum level of Dickkopf-1 (DKK-1) in postmenopausal females and to correlate its level with bone mineral density (BMD) and serum biochemical markers (calcium, phosphorus, and alkaline phosphatase).

## 2. Patients and Methods

 Sixty postmenopausal females were enrolled randomly for this study. Their age ranged from 45 to 52 years. The menopausal age ranged from 42 to 50 years. Those postmenopausal females were presenting to the rheumatology and rehabilitation outpatient clinic of Ain Shams University Hospitals complaining of mechanical low back pain (back muscle strain). 

 We excluded patients with osteoarthritis, patients with history of any chronic medical illness producing osteopenia/osteoporosis, thyroid or parathyroid disorder, history of chronic renal, hepatic or gastrointestinal disease including parasitic infestations, patients with prolonged intake of steroids, antiepileptic medication, females with history of cigarette smoking or alcohol abuse, Ca or vitamin D supplementation, hormone replacement therapy, or any drug affecting bone metabolism. 

 The study protocol was in accordance with Helsinki declaration of human rights and was approved by the local ethics committee. The written informed consent from each patient was obtained.

 All postmenopausal females were subjected to the following. 

(1) Full history taking and thorough physical examination including detailed musculoskeletal examination.

(2) Radiological investigations:

- Plain X-ray lumbosacral spine; anteroposterior and lateral views;
- bone mineral density (BMD) was measured by dual energy X-ray absorptiometry (DXA); GE Lunar prodigy at the femoral neck and axially at the lumbar spine (L1–L4) in the anterior and posterior projections. It was utilizing direct-digital detector technology. The patient lies on a large, flat padded table. To assess the spine, the patient's legs were supported on a padded box to flatten the pelvis and lower lumbar spine. To assess the hip, the patient's foot was placed in a brace that rotates the hip inward. In both cases, the detector was generating images on a computer monitor. The patient was asked to hold her breath for a few seconds, while the X-ray picture was being taken to prevent a blurred image;
- by DXA, results were recorded for each patient as *T* scores (difference in SD from the mean of a healthy young adult) [12]. Osteoporosis and osteopenia (low bone mass) were defined as *T* score less than −2.5 and *T* score between −1 and −2.5, respectively, [13]. Patients were divided according to bone mineral density into three groups: Osteoporosis, osteopenia (low bone mass), and normal DXA group.

(3) Laboratory investigations:

- complete blood count;
- erythrocyte sedimentation rate (ESR) using Westergren method;
- serum calcium and phosphorus by standard conventional assays;
- bone formation marker as bone-specific alkaline phosphates by Enzyme Linked Immunosorbent Assay that measures the level of the given ferment (ELISA: Tandem-MP Ostase, Beckman Coulter, USA);
- measurement of serum DKK-1 was done by ELISA for quantitative determination of DKK-1 in human serum. The kits were supplied from (Quantikine-R&D Systems Inc., USA). Serum was stored frozen at −80°C until measurement. Samples and standards were incubated in microplate wells and precoated with monoclonal anti-human DKK-1 antibody. After 120 minutes incubation at room temperature on a horizontal orbital microplate, shaing followed by washing step was done. Polyclonal anti-human DKK-1 antibody conjugated to horseradish peroxidase was added and incubated with the captured DKK-1. After another washing step, a substrate solution containing equal amount of stabilized hydrogen peroxide was added followed by 30 minutes incubation at room temperature on the benchtop protected from light. The reaction was stopped by addition of acidic solution. Absorbance of the resulting yellow product was measured at 450 nm against reference wave length of 540 nm. The absorbance is proportional to the concentration of DKK-1. A standard curve was constructed by plotting the mean absorbance against DKK-1 concentrations of standards on a log-log curve. Concentrations of unknown samples were determined using this standard curve.

(4) Statistical methods: statistical methods include IBM SPSS statistical software package (V. 19.0, IBM Corp., USA, 2010) that was used for data analysis. Data were expressed as range (minimum-maximum), mean, and standard deviation (SD) for quantitative measures and both number and percentage for categorical data. Comparison between two independent groups of numerical parametric data was done using the Student's *t*-test. One-way ANOVA test was used to compare between means of the three groups together. Pearson correlation test was done to study the correlation between two quantitative variables. Probability of error at <0.05 was considered significant and highly significant at <0.001. 

## 3. Results

 This study included 60 postmenopausal females; their age ranged from 45 to 52 years, with a mean of 49 ± 2.18 years. The menopausal age ranged from 42 to 50 years, with a mean of 45.88 ± 1.78 years. The postmenopausal duration ranged from 1 to 10 years with a mean of 3.23 ± 2.46 years. The serum DKK-1 levels in all postmenopausal females ranged from 1930 to 3530 pg/mL with a mean of 2581.5 ± 305.6 pg/mL.

 The serum calcium levels in all postmenopausal females ranged from 8.5 to 10.3 mg/dL with a mean of 9.3 ± 0.4 mg/dL. The serum phosphorus levels ranged from 2.7 to 4.1 mg/dL with mean of 3.5 ± 0.4 mg/dL, while the serum alkaline phosphatase levels ranged from 44 to 60.2 IU/L with mean of 54.8 ± 4.4 IU/L.

 Of the 60 postmenopausal females, fifteen had osteoporosis (25%); *T* score less than −2.5 in either lumbar spine or femur neck (group I), 27 (45%) had low bone mass; *T* score between −1 and −2.5 in either lumbar spine or femur neck (group II), and 18 postmenopausal females (30%) had normal *T* score in both lumbar spine and neck of femur and served as the control group. 

 In postmenopausal females with osteoporosis and low bone mass (group I and II), a highly significant increase was found in serum levels of DKK-1 and in duration of postmenopause compared to controls (*P* < 0.001). On the other hand, there was a significant decrease in serum calcium levels in postmenopausal females with osteoporosis and low bone mass versus controls (*P* = 0.025), while there was no significant difference between both groups regarding age, menopausal age, and serum levels of either phosphorus or alkaline phosphatase (*P* > 0.05) (Table 1).

 There was a highly statistically significant difference between the three groups (group I, group II, and controls) regarding the serum DKK-1 levels and the duration of menopause (*P* < 0.001), while serum calcium levels showed only significant difference between the three groups (*P* = 0.008). On the other hand, there was no a significant difference in age and serum levels of either phosphorus or alkaline phosphatase between the three groups (*P* > 0.05) (Table 2). DXA results in the three groups are shown in Table 3. 

**Figure 1:**
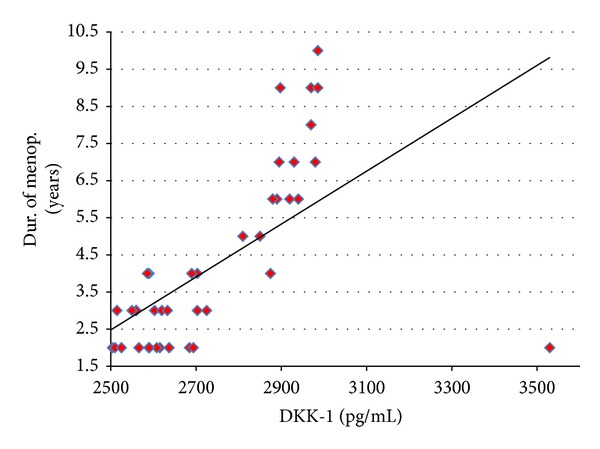
The positive correlation between serum DKK-1 levels and duration of postmenopause in both group I and group II.

 In both group I and group II (42 postmenopausal females), the duration of postmenopause correlated positively with serum levels of DDK-1 (*r* = 0.61, *P* < 0.001) (Figure 1) and correlated negatively with serum calcium levels (*r* = −0.41, *P* = 0.008). Also, the duration of menopause correlated negatively with lumbar *T* score (*r* = −0.86, *P* < 0.001) and with femur *T* score (*r* = −0.73, *P* < 0.001).

**Figure 2:**
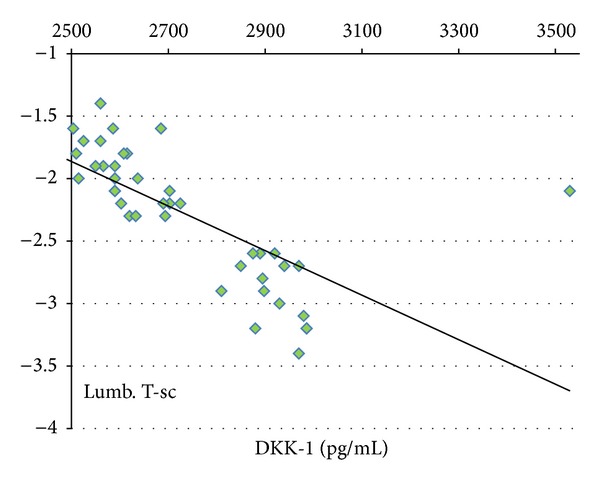
The negative correlation between serum DKK-1 levels and lumbar *T*-score in both group I and group II.

**Figure 3:**
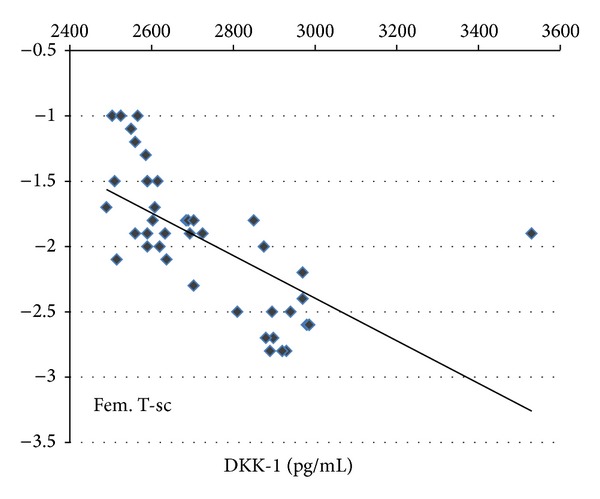
The negative correlation between serum DKK-1 levels and femur *T* score in both group I and group II.

 In addition, the serum DKK-1 levels correlated negatively with lumbar *T* score (*r* = −0.69, *P* < 0.001) (Figure 2) and with femur *T* score (*r* = −0.64, *P* < 0.001) (Figure 3), while there was no significant correlation between serum levels of either calcium, phosphorus, or alkaline phosphatase and both serum DKK-1 levels and the severity of osteoporosis as measured by *T* score (*P* > 0.05). 

## 4. Discussion

 Osteoporosis is widely recognized as an important public health problem because of the significant morbidity associated with its complications, mainly, fractures of the hip, spine and forearm [14]. In women, the loss of bone mass increases several years after postmenopause then decreases again. In postmenopausal women, the possibility of fractures should be kept in mind. The risk of fractures increases 1.5–3 times or more for a decrease of bone mineral density (BMD) by 1 standard deviation (SD) [15].

 There have been many cell signaling cascades linked to bone mass regulation. One pathway seen as critical for bone mass accrual, bone remodeling, and even fracture repair is the Wnt/*β*-catenin pathway. The Wnt/*β*-catenin pathway plays an important role in the development and maintenance of many tissues and organs. Disturbance in Wnt signaling is implicated in multiple disease processes. Wnt/*β*-catenin pathway acts as a major signaling cascade in bone biology [16].

 Dickkopf-1 (DKK1) is a soluble inhibitor of Wingless type Wnt/*β*-catenin signaling and is implicated in the regulation of osteoblast differentiation. It is a negative regulator of normal bone homeostasis in vivo [17]. DKK1 overexpression in osteoblasts causes osteopenia and inhibits fracture repair [18], while DKK1 activation in osteoblasts seems to participate in the pathogenesis of estrogen deficiency-mediated osteoporosis [19]. 

 The aim of this study was to assess serum level of Dickkopf-1 (DKK-1) in postmenopausal females and its correlation with bone mineral density (BMD) and serum biochemical markers in order to clarify if DKK1 has any role in the pathogenesis of osteoporosis.

 In this study, 25% of postmenopausal women showed osteoporosis (*T* score less than −2.5). The prevalence of osteoporosis in postmenopausal women aged from 47 to 60 years, based on WHO criteria using *T* scores, was 20.2% in the lumbar vertebrae. Similar results were obtained in postmenopausal women aged from 50 to 64 years in Germany, with a prevalence of osteoporosis in 23.3% [20]. Also, these results were similar to that reported by many researchers [21, 22]. 

 Sakondhavat et al. [23] and Li and Zhu [24] stated that the prevalence of osteoporosis increases proportionally with advancing age and duration of postmenopause in the lumbar vertebrae as well as the femoral neck. Zhai et al. [15] reported that with increasing age a significant loss of bone mass occurs in the vertebrae and femoral neck of postmenopausal women. The relationship between loss of bone mass and age is not linear, but quadratic. In contrast, Liu-Ambrose et al. [25] found no relationship between loss of bone mass and age. In the present study, the duration of postmenopause, not the age, appears to have a significant impact on the reduction of BMD in the lumbar vertebrae and femoral neck.

 In women entering menopause, there is a loss of bone mass of 1-2% annually for a period of 5–10 years. The loss of bone mass is particularly rapid in trabecular bone. Trabecular bone is affected by several factors, such as BMI, age, estradiol levels, and physical activity [26, 27]. 

 In postmenopausal women, there is a decrease in trabecular bone mass due to age and diminished estradiol levels. At the start of menopause, the mean loss in trabecular bone mass is reportedly between 1.8% and 2.3% in the vertebral column and between 1.0 and 1.4% in the pelvic bones. After 5 years of menopause the mean decrease in BMD is 7–10% in the vertebral column and 5–7% in the pelvic bones, thus, increasing the risk of fractures [28], while Finkelstein et al. [29] showed that the decline in BMD occurs significantly in late perimenopause and is extremely rapid in the first postmenopausal year. 

 Our results showed that DKK1 is increased in the serum of postmenopausal women with osteopenia and osteoporosis compared to controls (postmenopausal women with normal *T* score). The correlation observed between serum DKK1 and BMD of the lumbar vertebrae and femoral neck suggests that DKK1 is implicated, at least partially, in the pathogenesis of osteoporosis in postmenopausal women. This data suggests that DKK1 may play as a serological marker for bone mass evaluation in adults. The same results were reported in the study conducted by Butler et al. (2011) [16]. 

 We found a better negative correlation between serum DKK1 and lumbar BMD (*r* = −0.69, *P* < 0.001) compared to femur BMD (*r* = −0.64, *P* < 0.001). This may be explained by the severity of osteoporosis in the lumbar region observed in our cohort of patients. The same results were reported in the study among patients with thalassemia [30], while Anastasilakis et al. [31] reported no correlation between serum DKK-1 levels and BMD changes in postmenopausal women with established osteoporosis.

 Advancing age has recently been associated with changes in Wnt signaling, with decreased expression of Wnt-related proteins in the bone tissue. Furthermore, oxidative stress, a pivotal pathogenic factor of age-related bone loss and strength leading to a decrease in osteoblast number and function, is also linked to Wnt signaling inhibition [32]. This raises the issue that differences in serum DKK1 levels between the control and osteoporosis groups may be in fact an age-related phenomenon. This was subsequently disproved in our study due to two reasons: first, there was no significant difference between postmenopausal females with osteopenia and osteoporosis and controls regarding age; second, by using correlation and linear regression analysis in our data, there was no significant correlation between serum DKK1 levels and age. 

 The negative correlation between serum levels of DKK1 and alkaline phosphatase (an osteoblast product) is an indication that DKK1 plays a significant role in the biology of abnormal bone remodeling in postmenopausal osteoporosis [33]. This phenomenon has been already described in patients with myeloma [34] and patients with rheumatoid arthritis [11], where increased serum DKK1 directly impaired new bone formation through the reduction of osteoblast function and increased the production of RANKL and decreased the production of OPG by stromal cells and thus leading to increased bone resorption. However, this negative correlation was not recorded in our study which could be explained by the small number of patients in this study.

 Serum DKK-1 levels showed no significant correlation with serum levels of either calcium or phosphorus in this study. As far to our knowledge, very few data exist on this correlation. Therefore, further studies are warranted.

 Diagnosis and intervention should initially be aimed at postmenopausal women with the highest risk of osteoporosis. It is also essential to increase awareness of the implications of osteoporosis because in the majority of cases, it can be successfully diagnosed and treated to prevent fractures in postmenopausal women. DKK-1 blockade leads to overexpression of osteoprotegerin (OPG), a soluble inhibitor of osteoclastogenesis. So, DKK-1 inhibition may have a therapeutic approach in osteoporosis [11]. 

 Serum Dickkopf-1 levels may serve as a marker of bone metabolism and disease, although further standardization methods are required. Wnt pathway is currently a field of thorough investigation, but it is still far from being fully elucidated [35]. 

 However, the present study was not exempted from several limitations. Firstly, the study design was a cross-sectional type and thus was unable to prove that osteoporosis was the outcome of several risk factors. Secondly, the study did not collect data on lifestyle of postmenopausal women, as an influencing factor on the prevalence of osteoporosis.

In conclusion, postmenopausal women with significantly increased serum DKK-1 had more significant osteoporosis in lumbar spine and femur neck as detected by DXA. Prolonged duration of menopause and increased serum DKK-1 are important risk factors for the development and severity of osteoporosis. These results support the need for early prophylactic treatment in postmenopausal women. Also, it suggests that pharmacologic inhibition of DKK-1 may have a therapeutic approach in postmenopausal osteoporosis. 

## Figures and Tables

**Table 1:** Comparison between postmenopausal females with abnormal DXA results (groups I and II) and normal DXA results (controls) regarding demographic and laboratory data.

	Groups I and II *n* = 42		Controls *n* = 18		*P* value	Sig.
	Range	Mean ± SD	Range	Mean ± SD		
Age	47–52	49.95 ± 2.9	45–51	47.77 ± 1.8	*P* = 0.07	NS
Menopause age (yrs)	42–49	45.95 ± 1.76	44–50	45.72 ± 1.87	*P* = 0.66	NS
Dur. of postmenop. (yrs)	2–10	4.1 ± 2.4	1-2	1.0 ± 0.2	*P* < 0.001	HS
Ca (mg/dL)	8.5–10.2	9.2 ± 0.3	8.9–10.3	9.6 ± 0.5	*P* = 0.025	S
Ph. (mg/dL)	2.7–4.1	3.5 ± 0.43	2.7–4.1	3.5 ± 0.38	*P* = 0.86	NS
ALP (IU/L)	45.2–60.2	54.9 ± 4.1	44 –60.2	54.56 ± 5.1	*P* = 0.77	NS
DKK 1 (pg/mL)	2490–3530	2736.9 ± 206.87	1930–2395	2218.9 ± 148.26	*P* < 0.001	HS

Dur. of menop.: duration of menopause, Ca: calcium, Ph: phosphorus, ALP: alkaline phosphatase, DKK-1: Dickkopf, NS: nonsignificant, HS: highly significant, and S: significant.

**Table 2:** Laboratory data in groups I, group II, and controls. Comparison between three groups by ANOVA.

	Group I *n* = 15		Group II *n* = 27		Controls *n* = 18		*P* value	Sig.
	Range	Mean ± SD	Range	Mean ± SD	Range	Mean ± SD		
Ca (mg/dL)	8.5–9.6	9.1 ± 0.3	8.9–10.2	9.36 ± 0.3	8.9–10.3	9.6 ± 0.5	*P* = 0.008	S
Phosph. (mg/dL)	2.7–4.1	3.5 ± 0.47	2.7–4.1	3.5 ± 0.41	2.7–4.1	3.5 ± 0.38	*P* = 0.98	NS
Alk.ph (IU/L)	50.4–60.2	55.48 ± 2.9	45.2–60.2	54.67 ± 4.7	44–60.2	54.56 ± 5.1	*P* = 0.81	NS
DKK 1 (pg/mL)	2810–2986	2918.6 ± 53.73	2490–3530	2636 ± 190.52	1930–2395	2218.9 ± 148.26	*P* < 0.001	HS

Ca: calcium, Ph: phosphorus, ALP: alkaline phosphatase, DKK-1: Dickkopf, HS: highly significant, S: significant, and NS: Nonsignificant.

**Table 3:** DXA results in group I, group II, and controls.

	Group I *n* = 15		Group II *n* = 27		Controls *n* = 18	
	Range	Mean ± SD	Range	Mean ± SD	Range	Mean ± SD
Lumbar *T* score	−3.4–(−2.6)	−2.9 ± 0.26	−2.3–(−1.4)	−1.9 ± 0.25	−0.9–1.2	0.12 ± 0.77
Femur *T* score	−2.8–(−1.8)	−2.5 ± 0.29	−2.3–(−1.0)	−1.67 ± 0.36	−0.5–1.8	0.47 ± 0.71
